# Genotoxic activity of glyphosate and co-formulants in glyphosate-based herbicides assessed by the micronucleus test in human mononuclear white blood cells

**DOI:** 10.1016/j.toxrep.2025.102063

**Published:** 2025-06-03

**Authors:** Khadija Ramadhan Makame, Yazen Aljaber, Moustafa Sherif, Balázs Ádám, Károly Nagy

**Affiliations:** a Department Public Health and Epidemiology, Faculty of Medicine, University of Debrecen, Kassai Street 26/B, Debrecen H-4028, Hungary; b Doctoral School of Health Sciences, University of Debrecen, Kassai Street 26, Debrecen H-4028, Hungary; c Department of Anatomy and Embryology, Benha University, 13511, Al-Sahaa Street, Egypt; d Institute of Public Health, College of Medicine and Health Sciences, United Arab Emirates University, P. O. Box 17666, Al Ain, United Arab Emirates

**Keywords:** Glyphosate, Glyphosate-based herbicide, Adjuvant, Co-formulant, Genotoxicity, Micronucleus

## Abstract

Glyphosate-based herbicides (GBHs) are widely used and contribute to soil, water, and air contamination. Despite differing assessments of its carcinogenic potential, glyphosate toxicity may be enhanced by the co-formulants (adjuvants) used to improve its effectiveness. In this study, we investigated the genotoxic effects of glyphosate, alkyl dimethyl betaine (adjuvant A), and polyethoxylated tallow amine (adjuvant B) on human peripheral white blood cells using a cytokinesis block micronucleus (CBMN) assay. The experiments tested Glyphosate (0.1, 1, 10, and 100 μM) and adjuvants (at concentrations matching their levels in respective GBHs) in whole blood samples. The samples were exposed for 4 and 20 h with and without S9 metabolic treatment. The results showed that glyphosate and adjuvant A caused a statistically significant increase in the frequency of binucleated cells with micronuclei (BNMN%) only at 100 μM after 4-hour exposure without S9 treatment. Adjuvant B, however, induced a statistically significant increase in BNMN% starting at 1 μM after 4-hour exposure without S9 treatment. No significant effects were observed after 4 h of exposure with S9 or 20 h of exposure, with or without S9. The proliferation index (PI) showed no significant changes. This study concluded that the co-formulants in GBHs can induce genotoxic effects at low concentrations and short exposure times. This indicated that some surfactants in GBHs may be more toxic than glyphosate.

## Highlights

• Co-formulants in glyphosate herbicides are more genotoxic than glyphosate alone.
• Micronuclei increase was observed only at 4 h without S9 treatment.
• POEA causes significant DNA damage even at low concentrations.
• Surfactants in formulations may act synergistically to enhance toxicity.
• Findings highlight the need for stricter pesticide safety and regulation.

## Graphical Abstract

**Figure:**
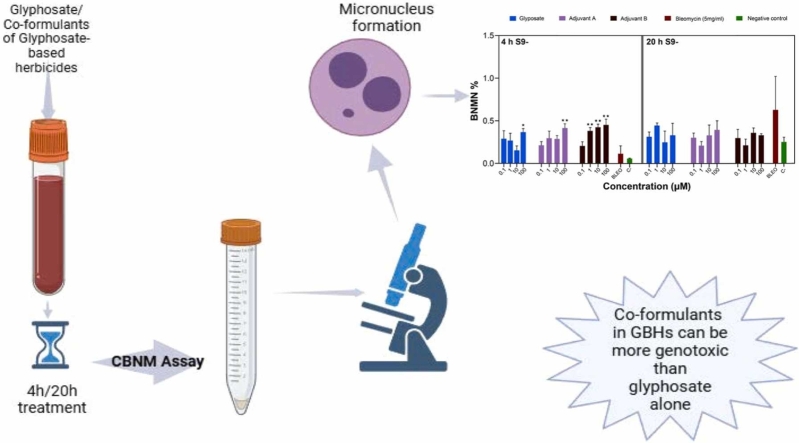

## 1 Introduction

The global use of glyphosate for agricultural and non-agricultural purposes has increased dramatically from roughly 67 thousand tons in 1995 to 826 thousand tons in 2014. It is expected to rise to 920 thousand tons by 2025 [1]. Its application is predicted to increase further owing to its use in glyphosate-tolerant genetically modified (GM) crop production systems [2]. Glyphosate is an effective weed killer with high diffusion capability, low acute toxicity, short half-life in the soil, and almost no volatility; it is considered environmentally friendly. However, in practice, glyphosate is never used independently but as an active ingredient in glyphosate-based herbicide formulations (GBHs). More than 2000 types of GBHs are commercially available worldwide [3], most of which have a glyphosate content of approximately 35–75 %, in the form of glyphosate acids or glyphosate salts [4]. These formulations are coupled with co-formulants or adjuvants to boost their herbicidal efficacy, increase permeability and solubility, and prevent degradation of the active ingredient [5]. These adjuvants can act as surfactants, antifoaming agents, or buffering agents to facilitate the coverage and penetration of glyphosate [6]. Although glyphosate is considered safe for animals [7], there is now sufficient scientific evidence to link glyphosate or GBHs to a wide range of health effects and disorders in humans and animals.

Several *in vitro* and *in vivo* studies suggest that glyphosate exposure can disrupt reproductive hormones, impair oocyte production, and increase embryo mortality in animals [8]. Research has shown oxidative stress and cellular damage caused by glyphosate and GBHs in various human and animal cells [9]. Glyphosate and GBHs have also been found to alter gut microbiota composition and metabolic function in species such as freshwater snails and rats. Notably, commercial GBH formulations caused more profound microbiome and metabolic disruptions than glyphosate alone, including interference with the shikimate pathway in rat guts [10], [11], [12]. Additional findings link glyphosate to neurotoxicity [13], [14], cardiovascular risks [15], organ damage [16], and interference with steroid hormone biosynthesis [17], with potential teratogenic and tumorigenic effects across species [18]. Studies show that glyphosate does not exhibit genotoxic effects on cultured human lymphocytes at low concentrations (20–40 µM), which are below environmental levels [19]. However, higher concentrations (above 200 µM) lead to slight DNA damage, and butterfly larvae exposed to glyphosate also showed increased genetic damage as indicated by higher micronuclei frequency [20].

Following the United States Environmental Protection Agency's (EPA) initial classification of glyphosate as unlikely to pose a cancer risk in 1991, which was subsequently reaffirmed in a 2017 review, and the International Agency for Research on Cancer's (IARC) categorisation of glyphosate as "probably carcinogenic to humans" (Group 2 A) in 2015 [21], [22], the scientific community has been intensively investigating the potential of glyphosate and glyphosate-based herbicides to induce DNA damage that could lead to mutations, cancer, developmental abnormalities, or other genetic disorders. The objective of these broader efforts by both regulatory bodies and independent researchers is to understand the potential genotoxic effects of glyphosate-based herbicides and address the differing conclusions reached by these groups. A comparison between glyphosate and a common GBH formulation Roundup (30 % glyphosate), revealed that even at low concentrations, Roundup can increase sister chromatid exchange frequency in human lymphocytes. It has also been found to trigger micronuclei formation in mouse bone marrow cells and cause oxidative damage to hepatocyte DNA within just a 24-hour exposure [23]. *In vivo* exposure of European eels *(Anguilla anguilla)* to Roundup®Ultra has also been demonstrated to cause oxidative DNA damage [24]. Additionally, Roundup® Full II formulation (66.2 % glyphosate) has also been demonstrated to cause significant DNA damage to the embryos of the crocodilian species *Caiman latirostris* when exposed to concentrations of ≥ 500 µg/mL (2957.8 µM) [25]. Károly and colleagues previously investigated the genotoxic potential of glyphosate and GBHs using two distinct methodologies, the comet assay and the cytokinesis block micronucleus assay, both employed in the analysis of human mononuclear white blood cells. The results of both assays consistently showed that glyphosate alone at concentrations below 1000 µM did not cause DNA damage. However, Roundup® Mega, Fozat 480, and Glyfos GBHs induce significant DNA damage in the presence or absence of S9 metabolic treatment [26], [27]. Similarly, the GBH formulation MON52276 induces alterations in gene expression in the rat liver by activating TP53, as well as increased apurinic/apyrimidinic (AP) site formation, implying that Roundup formulation can induce more biological changes associated with carcinogenesis than glyphosate alone. Nevertheless, it has been noted that glyphosate by itself can induce oxidative DNA damage in the livers of rats by modifying gene expressions, similar to what is seen with the MON52276 Roundup formulation [28]. Studies indicated that polyethoxylated tallow amine (POEA), a common surfactant in glyphosate-based herbicides, is 10–40 times more toxic to aquatic organisms and up to 1000 times more toxic to human cells than glyphosate alone [29]. An extensive body of research has demonstrated that GBHS exhibits heightened toxicity compared to glyphosate alone; however, debates are ongoing. These inconsistent findings, coupled with concerns regarding the potential health implications of glyphosate and GBHs, particularly concerning their genotoxic effects, underscore the need for further research. However, identifying the specific chemicals used as co-formulants is challenging because of patent protection, which complicates the comparison of results across studies [30]. This study investigated the potential of glyphosate and two typical co-formulants of glyphosate-based herbicides to induce genetic alterations in human mononuclear white blood (HMWB) cells.

## 2 Methods

The CBMN assay was performed on human mononuclear white blood cells derived from whole blood, following the standardized procedure described in the Organization for Economic Cooperation and Development (OECD) Guidelines for the Testing of Chemicals, In-Vitro Mammalian Cell Micronucleus Test [31], and C. Bolognesi and M. Fenech protocol [32], with slight adjustments (Fig. 2).

### 2.1 Chemicals

Analytical-grade glyphosate (CAS No: 1071–83–6) was obtained from the VWR International Kft (Debrecen, Hungary). The co-formulants analyzed included alkyl dimethyl betaine (EMPIGEN® BB detergent), a co-formulant present in Fozat 480, referred to as “adjuvant A” in this investigation, was purchased from Merck (Darmstadt, Germany), and polyethoxylated tallow amine (ROKAmin SR22), a co-formulant included in Glyfos, designated as “adjuvant B” in this analysis, which was kindly provided by the PCC Exol SA (Brzeg Dolny, Poland). In addition to these chemicals, RPMI 1640 medium and its supplements (VWR International; Leuven, Belgium), phytohemagglutinin (PHA) (Biosera; Cholet, France), human liver-derived metabolic activation system (S9 fraction) (Sigma-Aldrich Chemie GmbH, Heidelberg, Germany), cytochalasin-B (Cyt-B) (SERVA Electrophoresis GmbH, Heidelberg, Germany), and Giemsa staining solution (Merck, Darmstadt, Germany) were also used in this experiment.

### 2.2 Cell culture

Peripheral whole blood samples were obtained via venipuncture from three healthy, non-smoking, male volunteers aged between 35 and 37 years. The blood was collected in heparinized BD Vacutainer™ tubes (Becton, Dickinson and Company (BD); Plymouth, UK). None of the participants had been exposed to pesticides, mutagens, or carcinogens, and they provided informed consent before participating in the study. This study was approved by the Hungarian Ethical Committee for Medical Research (document 147–5/2019/EÜIG) and followed the ethical standards of the 2013 Declaration of Helsinki. Approximately 0.4 mL whole blood was added to a culture tube along with the appropriate volume of RPMI 1640 medium, supplemented with 10 % fetal calf serum (FCS), 2 mM/L-glutamine, 100 U/mL penicillin, 100 µg/mL streptomycin, and 250 ng/mL amphotericin. In addition, 1.5 % phytohemagglutinin (PHA) was used to stimulate lymphocyte proliferation. The final sample volume was 5 mL with the addition of the test chemicals, including the S9 mix. Cultures were prepared within 1 h of phlebotomy. To ensure that a significant proportion of lymphocytes actively proliferated and synchronized for subsequent chemical exposure, samples were incubated at 37 °C in a humidified incubator with 5 % CO₂ using loosely fitted lids. For the 4-hour exposure, cells were pre-incubated for 44-hours, followed by a 4-hour treatment, while for the 20-hour exposure, cells were pre-incubated for 28 h, followed by a 20-hour treatment**.**

### 2.3 Cell treatment

The cells were treated with different concentrations of glyphosate (0.1 µM, 1 µM, 10 µM, and 100 µM) or the two adjuvants. For adjuvants, concentration corresponds to their respective levels in the relevant GBH formulations. The tested concentrations for Adjuvant A, which is present at < 5 % w/w in Fozat 480, were equivalent to 5 × 10⁻⁷ %, 5 × 10⁻⁶ %, 5 × 10⁻⁵ %, and 5 × 10^−4^%. For Adjuvant B, which comprises 9 % w/w in Glyfos, the concentrations tested were 4.2 × 10⁻⁷ %, 4.2 × 10⁻⁶ %, 4.2 × 10⁻⁵ %, and 4.2 × 10⁻⁴ %, both corresponding to 0.1 µM, 1 µM, 10 µM, and 100 µM of glyphosate equivalents. The concentration range was optimized in accordance with the findings of previous studies [26], [27], [33], which indicated that the highest concentration that would not result in significant cell death was 100 µM. This concentration range was essential to avoid potential cytotoxic effects that could interfere with genotoxicity assessment. Cell viability was assessed before and after exposure to the tested chemicals using the trypan blue exclusion assay as previously described by Jauregui et al. [34]. In all cases, cell viability was greater than 95 %. Cell cultures were exposed to the test chemicals for 4 and 20 h. The experiment was performed in the presence and absence of human S9 fraction, in which 100 µL of the working S9 mix containing 10 % (v/v) S9 fraction composed of 8 mM MgCl_2_, 33 mM KCl, 100 mM sodium phosphate buffer (pH 7.4), 5 mM glucose-6-phosphate, and 4 mM NADP was used. The S9 fraction contains phase I and II metabolic enzymes that mimic the liver’s role in metabolizing xenobiotics. Their inclusion is crucial for identifying metabolic mutagens that require metabolic activation to exert genotoxic effects. Testing with and without S9 treatment allows differentiation between direct-acting and metabolically activated genotoxic substances in accordance with OECD guidelines for *in vitro* genotoxicity testing [31], [35]. In addition, 10 µL of 5 mg/mL bleomycin sulfate (BLEO) was used as the positive control. Bleomycin sulfate is a well-established genotoxic agent that induces DNA double-strand breaks and provides a reliable measure of the ability of our assay to detect DNA damage. The use of bleomycin as a positive control in genotoxicity assays has been supported by numerous studies [36], [37]. After 48 h of incubation, the samples were centrifuged for 10 min, and the supernatant was removed. The cells were resuspended in 4.9 mL of RPMI and 100 µL of 300 µg/mL cytochalasin B (Cyt-B) to inhibit cytokinesis. The cells were then incubated for an additional 20 h.

### 2.4 Cell harvesting

After 20 h incubation with Cyt-B, the samples were centrifuged for 10 min at a speed of 1500 rpm, the supernatant was removed, and the cells were resuspended in 0.075 M, KCl hypotonic solution at room temperature, mixed gently, and left at room temperature for 3 min to facilitate the lysis of red blood cells. After adding 400 µL of a pre-fixing solution (3:5 methanol: glacial acetic acid), the cell suspension was centrifuged at 1500 rpm for 10 min. The supernatant was discarded, and the cells resuspended in 5 mL of fixing solution (5:1 methanol to glacial acetic acid), gently mixed, and left for 30 min. This centrifugation step was repeated three times. Finally, the cells were resuspended in 500 µL of the fixing solution and placed on slides for staining.

### 2.5 Slide preparation and staining

The cell suspension was dropped onto iced slides, dried at room temperature, and stained with 3 % Giemsa in Sorensen’s buffer (pH 6.8) for 5 min. After rinsing with 1.5 % Sorensen buffer, slides were dried and mounted using Eukitt glue.

### 2.6 Slide scoring

**Fig. 1:**
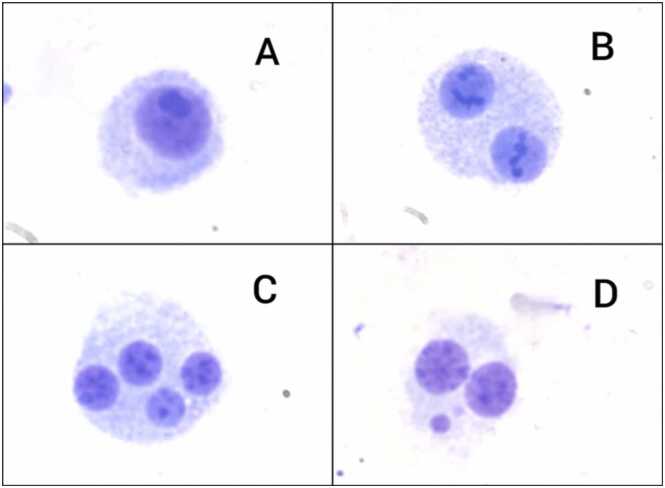
Examples of a mononucleated cell (A), binucleated cell (B), multinucleated cell (C), and binucleated cell with micronucleus (D), as seen under high-resolution optical microscope (ZEISS®, Germany) at 1000x magnification.

The slides were blindly coded, and visual assessments was performed using a high-resolution optical microscope equipped with a Zeiss Axiocam 503 mono digital camera (ZEISS®, Germany) at a magnification of 400x. Two individuals conducted slide scoring; each experiment comprised two replicates, with each evaluator independently scoring different replicates of the same experiment. The final score for each experiment was determined by averaging the results of both replicates. A total of 2000 binucleated cells (1000 per slide) were scored for each experimental point. The proliferation index (PI) was established by counting a minimum of 1000 cells containing either one nucleus (mononucleated), two nuclei (binucleated), or more than two nuclei (multinucleated) (examples of the cells as seen in Fig. 1), using the formula:

PI= [(nMONO)+ 2(nBN)+ 3(nMULTI)]/N

Where:

**Fig. 2:**
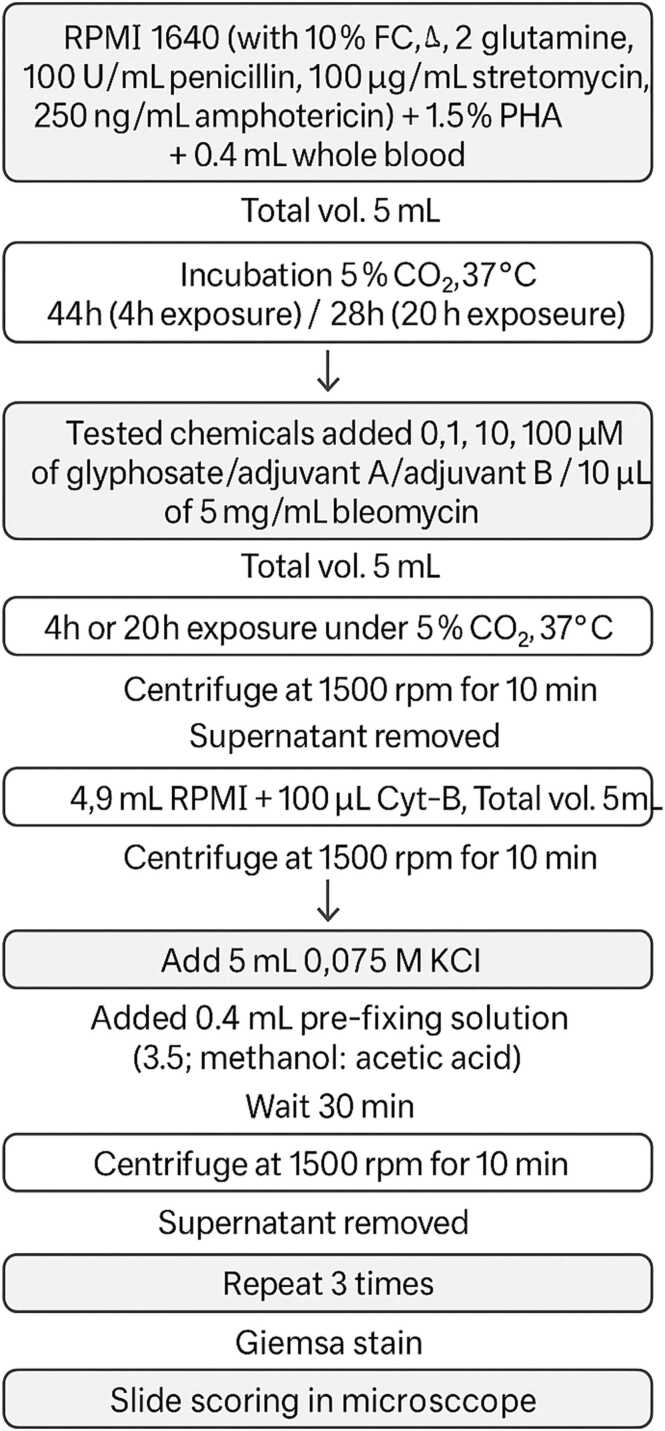
Schematic representation of the cytokinesis-block micronucleus (CBMN) assay protocol used for evaluating the genotoxicity of the test agents in human mononuclear white blood (HMWB) cells.

PI is the proliferation index,

nMONO is the number of mononuclear cells,

nBN is the number of binuclear cells,

nMULTI is the number of multinucleated cells, and

N is the total number of cells counted.

Micronuclei frequency (BNMN%) was determined by calculating the ratio of binucleated cells containing micronuclei (BNMN) to the total number of binucleated cells. Micronuclei identification followed the guidelines established by Fenech et al. [32].

### 2.7 Statistical analysis

Statistical analyses were performed using R (R Core Team 2024, 4.4.2 release) and RStudio (RStudio 2024.09.1 +394 “Cranberry Hibiscus” release). Data are presented as mean ± standard error of the mean (SEM) derived from three independent experiments, with two replicates. The frequencies of binucleated cells with micronuclei (BNMN%) and the proliferation index (PI) for various treatment concentrations were statistically compared to those of untreated cells using one-way ANOVA and Dunnett’s post-hoc test. The significance level was set at 5 % (p-value < 0.05).

## 3 Results

**Fig. 3:**
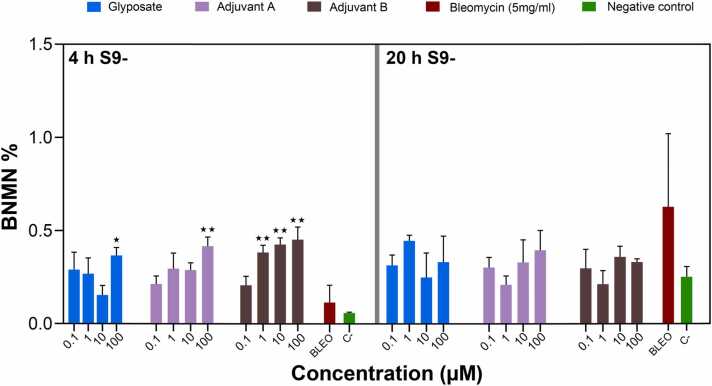
Percentage of binucleated cells with micronuclei (BNMN%) observed after a 4 and 20-hour exposures to varying concentrations of glyphosate and two co-formulants, adjuvant A and adjuvant B, without S9 treatment (S9-). Data points represent the mean ± standard error of the mean (SEM) from three independent experiments. A statistically significant (* p < 0.05, ** p < 0.01) increase was analyzed by comparing the frequency of binucleated cells with micronuclei induced by various doses of test chemicals to the background level of untreated cells by ANOVA with Dunnett’s post hoc test.

**Fig. 4:**
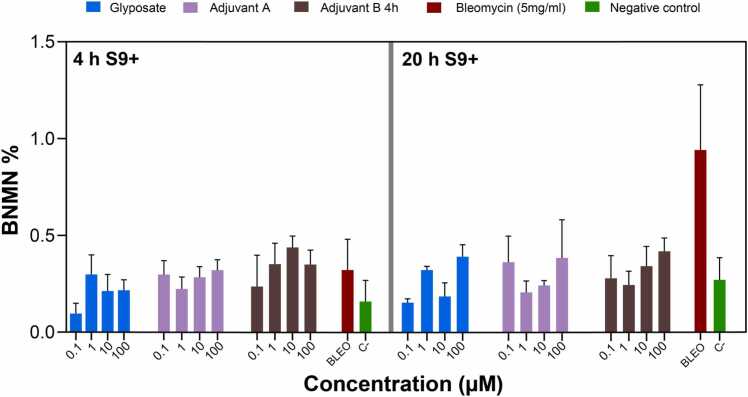
Percentage of binucleated cells with micronuclei (BNMN%) observed after a 4 and 20-hour exposures to varying concentrations of glyphosate and two co-formulants, adjuvant A and adjuvant B, with S9 treatment (S9+). Data points represent the mean ± standard error of the mean (SEM) from three independent experiments. A statistically significant increase was analyzed by comparing the frequency of binucleated cells with micronuclei induced by various doses of test chemicals to the background level of untreated cells by ANOVA with Dunnett’s post hoc test.

Genotoxicity, indicated by an increase in binucleated cells with micronuclei (BNMN%), varied with the concentration of glyphosate and co-formulants after 4- and 20-hour exposures, with and without S9 treatment. Without S9 treatment, a statistically significant increase in BNMN% was observed after 4 h of exposure to 100 µM glyphosate (0.218 % ± 0.055 %, p < 0.05) and 100 µM adjuvant A (0.321 % ± 0.055 %, p < 0.01), indicating elevated genotoxic effects at higher concentrations. Treatment with adjuvant B induced a statistically significant increase at concentrations of 1 µM (0.352 % ± 0.108 %, p < 0.01), 10 µM (0.439 % ± 0.060 %, p < 0.01), and 100 µM (0.350 % ± 0.075 %, p < 0.01). In comparison to the results of 4-hour exposure, BNMN% values observed after 20-hour treatment were consistently lower for all agents and concentrations tested, both with and without S9 treatment; however, these decreases were not statistically significant (p > 0.05 for all comparisons) (Fig. 3, Fig. 4). High proliferation index (PI) levels were observed for all treated agents (Table 1, Table 2). Comparison of the PI observed in the presence and absence of S9 fractionbetween the concentrations of each chemical did not show statistically significant differences.

**Table 1:** Proliferation index (PI) of HMWB cells at various concentrations after a 4-hour exposure to glyphosate, adjuvant A, and adjuvant B, with and without S9 metabolic treatment.

**Concentration (µM)**	**Glyphosate**		**Adjuvant A**		**Adjuvant B**	
	**With S9**	**Without S9**	**With S9**	**Without S9**	**With S9**	**Without S9**
**0.1**	1.594 ± 0.039	1.737 ± 0.101	1.686 ± 0.030	1.638 ± 0.039	1.672 ± 0.067	1.588 ± 0.082
**1**	1.633 ± 0.039	1.732 ± 0.050	1.637 ± 0.041	1.615 ± 0.101	1.623 ± 0.048	1.714 ± 0.047
**10**	1.791 ± 0.035	1.757 ± 0.059	1.796 ± 0.058	1.733 ± 0.051	1.642 ± 0.044	1.729 ± 0.061
**100**	1.737 ± 0.013	1.824 ± 0.077	1.839 ± 0.035	1.696 ± 0.058	1.740 ± 0.054	1.722 ± 0.033
	**With S9**	**Without S9**				
**mg/mL**	1.707 ± 0.023	1.619 ± 0.025				
**Negative control**	1.676 ± 0.057	1.614 ± 0.025

**Table 2:** Proliferation index (PI) of HMWB cells at various concentrations after a 20-hour exposure to glyphosate, adjuvant A, and adjuvant B, with and without S9 metabolic treatment.

Concentration (µM)	Glyphosate		Adjuvant A		Adjuvant B	
	**With S9**	**Without S9**	**With S9**	**Without S9**	**With S9**	**Without S9**
0.1	1.595 ± 0.119	1.601 ± 0.089	1.654 ± 0.081	1.705 ± 0.027	1.567 ± 0.050	1.649 ± 0.060
1	1.651 ± 0.102	1.599 ± 0.068	1.654 ± 0.074	1.678 ± 0.059	1.676 ± 0.070	1.706 ± 0.088
10	1.607 ± 0.132	1.662 ± 0.073	1.715 ± 0.034	1.689 ± 0.055	1.658 ± 0.043	1.771 ± 0.087
100	1.633 ± 0.028	1.686 ± 0.077	1.767 ± 0.082	1.751 ± 0.074	1.647 ± 0.043	1.695 ± 0.085
	**With S9**	**Without S9**				
Bleomycin 5 mg/mL	1.606 ± 0.034	1.672 ± 0.030				
Negative control	1.617 ± 0.041	1.703 ± 0.091				

The data are presented as the mean ± standard error of the mean (SEM) from three independent experiments. Statistical significance for the PI was assessed by comparing PI values for various doses of glyphosate, adjuvant A, and adjuvant B against untreated cells using ANOVA followed by Dunnett’s post hoc test.

The data are presented as the mean ± standard error of the mean (SEM) from three independent experiments. Statistical significance for the PI was assessed by comparing PI values for various doses of glyphosate, adjuvant A, and adjuvant B against untreated cells using ANOVA followed by Dunnett’s post hoc test.

## 4 Discussion

This study evaluated the genotoxic effects of glyphosate, alkyl dimethyl betaine (adjuvant A), and polyethoxylated tallow amine (adjuvant B) on human peripheral white blood cells using the cytokinesis block micronucleus (CBMN) assay, which is a widely validated method for assessing genotoxic biomarkers. [31], [32]. Glyphosate is extensively employed in agricultural practices in formulations that incorporate co-formulants to enhance their efficacy [5], [38], [39]. This has given rise to concerns regarding the primary source of toxicity, whether attributable solely to the active ingredient or co-formulants, which contribute substantially to overall toxicity. [30], [39], [40].

Our study measured the key indicators of genotoxic potential, including micronucleus frequency (BNMN%) and proliferation index (PI) [41], [42], [43], [44]. Micronucleus formation has been identified as a marker of chromosomal damage, with the potential to cause mutations and an increased risk of cancer development. An elevated micronucleus frequency has been shown to reflect genomic instability. It is a standard tool used in toxicological and environmental studies to evaluate mutagenic and carcinogenic risks [45], [46]. The dose–response curves (Fig. 3, Fig. 4) suggested that genotoxicity did not increase linearly with concentration for any tested agents. Depending on the concentration and exposure time, glyphosate showed a lower or comparable micronucleus frequency than adjuvants A and B. When exposed to glyphosate, cells showed a statistically significant increase in micronucleus frequency only at a concentration of 100 µM after 4-hour exposure without S9, which is consistent with the findings of Nagy et al., who observed similar effects after 20-hour exposure with and without S9 treatment [27]. Previous studies have also reported variations in micronucleus frequency following glyphosate exposure. For example, an *in vitro* study using cultured human lymphocytes reported a significant increase in micronucleus frequency at lower concentrations (0.295 μM to 2.95 μM) after a 44-hour incubation [47]. Also, Kasuba et al. observed an increased frequency of micronuclei in HepG2 cells exposed to glyphosate at concentrations relevant to the Acceptable Daily Intake (ADI), Residential Exposure Limit (REL) and Occupational Exposure Limit (OEL), with significant effects observed at concentrations of 2.9 μM, 17.2 μM and 20.7 μM after 4-hours of exposure, followed by a decline after 24-hours [48]. It is essential to recognise that variations in micronucleus formation across different studies could be attributed to the unique characteristics of the cell types used. HepG2 cells from hepatocellular carcinoma showed chromosomal instability and reduced DNA repair capacity. These factors may explain why they tend to produce a higher frequency of micronuclei at lower concentrations than primary mononuclear white blood cells [49]. In addition, previous studies have reported that glyphosate and its formulations have genotoxic effects on specific cancer cell lines, such as HEC1A and MDA-MB-231, which may be influenced by oestrogen responsiveness and the presence of adjuvants or impurities in commercial formulations [50]. Alkyl dimethyl betaines, identified in this study as adjuvant A, function as surfactants frequently employed in formulations such as Fozat 480. According to the European Chemicals Agency (ECHA), alkyl dimethyl betaine has been associated with adverse effects, such as severe eye damage, skin burns, and irritation. However, there is no evidence of carcinogenicity or genotoxicity. Evaluation of the genotoxicity of alkyl dimethyl betaines has primarily relied on *in vitro* methods, including the bacterial reverse mutation assay (Ames test), which assesses mutagenicity across five strains of *Salmonella typhimurium*, and the chromosome aberration test, which evaluates the clastogenic effects in mammalian cells (Chinese hamster ovary cells). A CHO/HGPRT mutation assay was also used to detect gene mutations in mammalian cells. The results from these assays consistently demonstrated no genotoxic effects, irrespective of the presence or absence of S9 metabolic treatment [51]. In contrast, the present findings revealed a significant increase in the frequency of binucleated cells with micronuclei induced by adjuvant A at a concentration of 100 μM following a 4-hour exposure without S9 treatment. These results are consistent with those reported by Nagy et al., further supporting their observations, who reported a statistically significant increase in micronucleus frequency with Fozat 480 at the same concentration of 100 μM after a 4-hour exposure without S9 treatment. This finding indicates a contribution to the overall genotoxic effect [27]. However, significant results were also observed by Nagy et al. at the same concentration with S9 treatment and both 10 μM and 100 μM concentrations under conditions with and without S9 treatment after a 20-hour exposure, although these findings were not replicated in the present study [27].

Our study revealed a statistically significant increase in MN frequency following a 4-hour exposure to adjuvant A at 100 μM without S9 treatment, suggesting that the parent compound (alkyl dimethyl betaine) can exert direct genotoxic effects. This observation aligns with the findings of Nagy et al., who reported similar MN induction under identical conditions using Fozat 480, supporting the hypothesis that co-formulants within GBHs contribute substantially to genotoxicity, independent of S9 treatment. [27]. Although Nagy et al. also observed significant MN formation with and without S9 treatment after 20-hour exposures, our study could not reproduce these results. This discrepancy may arise from differences in the formulation composition, cell sensitivity or exposure protocols.

The absence of MN induction in response to S9 treatment in our experiment further supports the interpretation that the S9 metabolic fraction may act to detoxify, rather than activate, the tested compounds. According to OECD Test Guideline No. 487, metabolic activation systems such as the S9 mix are used to simulate mammalian metabolism *in vitro*. In our study, the absence of significant genotoxicity in the presence of S9 suggests that the test substances are unlikely to be metabolized into genotoxic compounds under the conditions used [31]. These results agree with broader findings in genotoxicity research, highlighting that certain compounds exert genotoxicity only in their parent form [52] and that metabolic enzymes may reduce their potency [31], [53]. Therefore, our findings emphasise the importance of evaluating chemical genotoxicity, both with and without metabolic activation, especially for mixtures such as GBHs, where co-formulants (e.g. surfactants and adjuvants) may be directly responsible for adverse effects. From an environmental health perspective, this outcome raises significant concerns for cell types with limited metabolic capacity, such as peripheral blood lymphocytes, skin cells, and reproductive cells, that may be particularly vulnerable to direct-acting genotoxins. This is especially important for exposure scenarios involving dermal absorption or inhalation, where chemicals may bypass hepatic metabolism and reach the portal of entry and the systemic circulation in their active form. Consequently, individuals working in agriculture, pesticide handling, and chemical manufacturing may face an increased risk of DNA damage and related health effects [54]. Although glyphosate is not highly persistent in the environment, several co-formulants and surfactants used in GBHs exhibit high environmental persistence and toxicity. This concerns the potential long-term ecotoxicological effects, particularly on aquatic organisms, soil invertebrates, and microbial communities, which often lack effective metabolic detoxification mechanisms. Prolonged exposure can induce genotoxic stress, reproductive impairment, population decline, and broader disruptions to ecosystem stability [3], [55], [56]. These findings underscore the need for regulatory frameworks that consider non-metabolising cell types and realistic exposure pathways. However, current risk assessments relying solely on S9-activated systems may underestimate the hazards of direct-acting genotoxins within complex formulations, such as GBHs [53], [57]. Hence, it is important to routinely apply genotoxicity assays both with and without metabolic activation, and to individually assess GBH co-formulants in addition to their active ingredient glyphosate [2], [22].

Polyethoxylated tallow amine (POEA), designated as adjuvant B in this study, is a surfactant that is frequently employed in glyphosate-based herbicides, including glyphosate, to enhance the objective of improving their capacity to penetrate plant tissues and distribute more efficiently [58]. This chemical has been proscribed in the European Union on the basis that it has been demonstrated to exert a toxic effect on humans and aquatic organisms [59]. The present study demonstrated a statistically significant increase in the frequency of binucleated cells with micronuclei following exposure to adjuvant B (POEA) at concentrations of 1 μM, 10 μM, and 100 μM after a 4-hour treatment without S9 treatment. This finding demonstrated a dose-dependent increase in micronucleus frequency, which exceeded the effects of glyphosate and adjuvant A. These results are in agreement with those reported by Nagy et al., who reported a significant increase in the percentage of binucleated cells with micronuclei (BNMN%) induced by glyfos at concentrations of 10 μM and 100 μM after 4-hour exposure [27]. An *in vivo* assessment was conducted to examine the relative contribution of the active ingredient glyphosate and surfactant polyethoxylated tallow amine (POEA) to the genotoxicity of a commercial formulation in *Anguilla anguilla* fish. This assessment revealed clear, evident, and apparent genotoxic effects, including extensive DNA breaks and oxidative DNA damage. The findings indicated that POEA induced a significantly greater extent of DNA damage than glyphosate and the commercial mixture, suggesting an additive impact on toxicity within the mixture, indicating that the combined exposure results in a level of toxicity equivalent to the sum of the individual effects. Furthermore, both components are independent contributors to genotoxicity [60]. A further study investigating DNA damage in human lung A549 cells exposed to Roundup® and POEA found a significant increase (p < 0.01) in tail length, tail DNA percentage, and tail moment in the comet assay for both the POEA and Roundup® treatment groups. Consequently, the study concluded that POEA, rather than the active ingredient glyphosate in Roundup®, was predominantly responsible for the observed genotoxicity [61]. Moreover, an *in vivo* evaluation using zebrafish larvae and an *in vitro* study utilizing rainbow trout gonad-2 (RTG-2) cells assessed the impact of a glyphosate-based commercial herbicide, its constituent components, and its metabolite AMPA on non-target aquatic organisms using a comet assay. The results revealed a significant genotoxic effect at concentrations of 0.4 mg/L (0.8 μM) and 1.6 mg/L (3.2 μM), respectively. These findings are in close agreement with the observations recorded and further demonstrate the deleterious effects of these substances, even at low concentrations [62].

Another interesting observation of this study is that significant micronucleus formation was observed only at 4-hour and not 20-hour of exposure. One possible explanation for this could be that short-term exposure, up to 4-hours, may cause acute genotoxic stress that triggers prompt cellular responses before adaptive mechanisms or DNA repair pathways are activated. In contrast, during the prolonged exposure of 20-hours, cells have more time to activate DNA repair processes or to undergo cell cycle arrest and apoptosis, thereby reducing the number of damaged cells that progress to cytokinesis and contribute to the development of MN. This phenomenon has also been observed in other studies where genotoxic effects at early time points were due to transient DNA damage [63]. Additionally, prolonged exposure to toxic compounds can lead to increased cell death or reduced proliferation, which limits the potential for MN to develop. Some studies with glyphosate and GBHs have also reported time-dependent differences in genotoxic responses, where effects being more detectable at shorter exposures of approximately 20 min [64] and 7 h [65]. Therefore, significant MN induction at 4-hours may reflect a peak of genotoxic activity before compensatory cellular mechanisms are engaged These findings suggest that timing is a critical factor in genotoxicity assays and highlight the importance of including multiple exposure durations to capture different aspects of chemically induced cellular damage.

In our study, the proliferation index (PI) exhibited no substantial alterations in response to 4-hour and 20-hour treatments, and no significant differences were observed irrespective of the presence or absence of S9 fraction. These results are consistent with the findings reported by Nagy et al. [26], [27] and are further supported by an earlier study conducted by Santovito et al. [62]. However, another study reported a notable decrease in the proliferation index for glyphosate at concentrations of 0.1 μg/mL (0.59 μM), 0.25 μg/mL (1.48 μM), and 0.5 μg/mL (2.96 μM) compared to the control group [47]. These findings suggest that toxins do not affect cell division. Nevertheless, Santovito's recent experiments challenged this assumption, raising questions regarding the potential variability in cellular responses. It is also possible that the increased proliferation index was the result of severe oxidative DNA damage, such as pyrimidine oxidation. Such damage leads to genomic instability and alterations in membrane integrity, as highlighted by Guilherme et al. [24]. Alternatively, the increased proliferation index could be the result of oxidative damage caused by increased superoxide dismutase (SOD), as highlighted by Makame et al. [33] and Mesnage et al. [40].

Few studies have directly compared the genotoxic effects of primary glyphosate-based herbicide formulations with their respective co-formulants or surfactants. The present study's findings support the hypothesis that co-formulants play a critical role in enhancing toxicity and genotoxicity, particularly at lower concentrations. Our findings suggest that surfactants may not only exacerbate the toxicity of the active ingredient, but could also serve as the primary drivers of toxic effects. The observed increase in micronucleus frequency and proliferation index has significant implications for human health, as these indicators reflect elevated genotoxic stress and potential genomic instability, both of which are associated with an increased risk of mutagenesis and carcinogenesis.

We acknowledge some limitations in this study as, firstly, the use of samples from different donors throughout the experiment introduces variability due to individual genetic differences and lifestyle factors. Second, although concerted efforts were made to select participants without direct exposure to pesticides or high-risk environments, it is impossible to completely rule out the presence of trace amounts of pesticides because of widespread environmental and dietary contamination. Finally, variability among observers during manual microscope assessments and cell scoring by two individuals has the potential to influence the results. This potential limitation could have been mitigated by employing automated systems, such as high-resolution digital microscopes equipped with advanced scanning capabilities and flow cytometry, to ensure greater accuracy and consistency in data collection. In addition, using additional genotoxicity assays, such as the comet or γH2AX assays, could provide a more comprehensive assessment of the DNA-damaging potential of glyphosate-based herbicides and their co-formulants.

## 5 Conclusion

The present study demonstrates that co-formulants in GBHs induce significant genotoxic effects, even at low concentrations and after short exposure. These findings provide substantial evidence that surfactants play a considerable role in the overall toxicity of GBHs, often exhibiting greater toxicity than the active ingredients. This study is among the few to use the micronucleus assay, a reliable predictor of potential carcinogenicity, to assess the genotoxicity of these substances. Polyethoxylated tallow amine (POEA) has been shown to exhibit high toxicity in previous and present study. These findings emphasise the need for governments to implement stringent mitigation measures, such as those adopted by the European Union, to phase out POEA and explore safer alternatives. The heightened genotoxic potential exhibited by adjuvants such as POEA compared to glyphosate alone prompts critical concerns regarding the safety evaluation of commercial formulations. However, global phase-out of POEA should not automatically imply that all alternative surfactants are inherently safe. Although quaternary ammonium compounds and other co-formulants have been proposed as safer substitutes [66], [67], emerging evidence has highlighted the potential toxicological concerns associated with these alternatives. For instance, formulations such as Roundup MON 52276, which contain propoxylated quaternary ammonium compounds, have been shown to disrupt the gut microbiome and raise long-term health concerns [12], [68]. Moreover, studies by Ferguson et al. and Panzacchi et al. have demonstrated that some co-formulants, such as those in Roundup Probio (e.g., alkylpolyglycoside and nitroryl), can reduce HepG2 cells viability more significantly than glyphosate alone [69], [70]. These findings emphasise the critical need for comprehensive toxicological assessments of all GBH co-formulants before their approval and widespread use rather than assuming safety by default. Adjuvants, which are frequently presumed to be inert, can independently induce substantial genetic damage and may act synergistically with active ingredients, thereby amplifying the toxic effects. This study underscores the pressing need for more comprehensive safety assessment of pesticide formulations. Such evaluations should encompass the potential risks associated with all components, including co-formulants, as opposed to a narrow focus on active ingredients, to facilitate a more comprehensive understanding and effective mitigation of the risks posed by these formulations to human health.

## CRediT authorship contribution statement

**Károly Nagy:** Writing – review & editing, Writing – original draft, Visualization, Validation, Supervision, Software, Resources, Project administration, Funding acquisition, Conceptualization. **Yazen Aljaber:** Software, Methodology, Investigation, Formal analysis, Data curation. **Khadija Ramadhan Makame:** Writing – original draft, Visualization, Software, Resources, Investigation, Formal analysis, Data curation, Conceptualization. **Balázs Ádám:** Writing – review & editing, Conceptualization. **Moustafa Sherif:** Writing – review & editing, Software, Methodology, Formal analysis.

## Informed consent statement

All blood donors provided written informed consent prior to blood sample collection.

## Ethical statement

Institutional Review Board Statement: This study adhered to the principles outlined in the Declaration of Helsinki and was approved by the Scientific and Research Ethics Committee of the University of Debrecen and the Ethical Board of the National Public Health Centre (NPHC) of Hungary on 12 February, 2019 (Approval No.147–5/2019/EÜIG).

## Funding

This study was supported by the János Bolyai Research Scholarship of the 10.13039/501100003825Hungarian Academy of Sciences and the Stipendium Hungaricum Scholarship Program PhD grant.

## Declaration of Competing Interest

The authors declare the following financial interests/personal relationships which may be considered as potential competing interests: Karoly Nagy reports financial support was provided by Hungarian Academy of Sciences. Khadija Ramadhan Makame reports financial support was provided by Stipendium Hungaricum Scholarship Programme PhD grant. If there are other authors, they declare that they have no known competing financial interests or personal relationships that could have appeared to influence the work reported in this paper.

## Data Availability

Data will be made available on request.

## References

[1] Maggi F., Tang F.H.M., la Cecilia D., McBratney A. (2019). PEST-CHEMGRIDS, global gridded maps of the top 20 Crop-Specific pesticide application rates from 2015 to 2025. Sci. Data.

[2] Benbrook C.M. (2016). Trends in glyphosate herbicide use in the United States and globally. Environ. Sci. Eur..

[3] Klátyik S., Simon G., Oláh M., Mesnage R., Antoniou M.N., Zaller J.G., Székács A. (2023). Terrestrial ecotoxicity of glyphosate, its formulations, and Co-Formulants: evidence from 2010–2023. Environ. Sci. Eur..

[4] Cuhra M., Bøhn T., Cuhra P. (2016). Glyphosate: too much of a good thing?. Front. Environ. Sci..

[5] Altmanninger A., Brandmaier V., Spangl B., Gruber E., Takács E., Mörtl M., Klátyik S., Székács A., Zaller J.G. (2023). Glyphosate-Based herbicide formulations and their relevant active ingredients affect soil springtails even five months after application. Agriculture.

[6] Pesticide Action Network Europe Pesticide Co-Formulants: Hidden Toxins Remain Under the Radar Brussels, Belgium, 2023. Available online: 〈https://www.pan-europe.info/facsheets/pesticide-co-formulants-hidden-toxins-remain-under-radar〉 (accessed on 6 May 2025).

[7] Kier L.D., Kirkland D.J. (2013). Review of genotoxicity studies of glyphosate and Glyphosate-based formulations. Crit. Rev. Toxicol..

[8] Jarrell Z.R., Ahammad M.U., Benson A.P. (2020). Glyphosate-Based herbicide formulations and reproductive toxicity in animals. Vet. Anim. Sci..

[9] Gasnier C., Dumont C., Benachour N., Clair E., Chagnon M.C., Séralini G.E. (2009). Glyphosate-based herbicides are toxic and endocrine disruptors in human cell lines. Toxicology.

[10] Bao Y., He X., Zhai Y., Shen W., Jing M., Liu Y., Yang H., Chen L. (2024). Effects of Glyphosate-based herbicide on gut microbes and hepatopancreatic metabolism in pomacea canaliculata. Ecotoxicol. Environ. Saf..

[11] Mesnage R., Panzacchi S., Bourne E., Mein C.A., Perry M.J., Hu J., Chen J., Mandrioli D., Belpoggi F., Antoniou M.N. (2022). Glyphosate and its formulations roundup bioflow and RangerPro alter bacterial and fungal community composition in the rat caecum microbiome. Front. Microbiol..

[12] Mesnage R., Teixeira M., Mandrioli D., Falcioni L., Ibragim M., Ducarmon Q.R., Zwittink R.D., Amiel C., Panoff J.M., Bourne E. (2021). Multi-omics phenotyping of the Gut-Liver axis reveals metabolic perturbations from a Low-Dose pesticide mixture in rats. Commun. Biol..

[13] Cattani D., de Liz Oliveira Cavalli V.L., Heinz Rieg C.E., Domingues J.T., Dal-Cim T., Tasca C.I., Mena Barreto Silva F.R., Zamoner A. (2014). Mechanisms underlying the neurotoxicity induced by Glyphosate-Based herbicide in immature rat hippocampus: involvement of glutamate excitotoxicity. Toxicology.

[14] Malhotra R.C., Ghia D.K., Cordato D.J., Beran R.G. (2010). Glyphosate-surfactant herbicide-induced reversible encephalopathy. J. Clin. Neurosci..

[15] Gress S., Lemoine S., Séralini G.E., Puddu P.E. (2015). Glyphosate-based herbicides potently affect cardiovascular system in mammals: review of the literature. Cardiovasc. Toxicol..

[16] Abdelmagid A.D., Said A.M., Abd El-Gawad E.A., Shalaby S.A., Dawood M.A.O. (2023). Glyphosate-Induced liver and kidney dysfunction, oxidative stress, immunosuppression in Nile tilapia, but ginger showed a protection role. Vet. Res. Commun..

[17] Walsh L.P., McCormick C., Martin C., Stocco D.M. (2000). Roundup inhibits steroidogenesis by disrupting steroidogenic acute regulatory (StAR) protein expression. Environ. Health Perspect..

[18] Mesnage R., Defarge N., Spiroux de Vendômois J., Séralini G.E. (2015). Potential toxic effects of glyphosate and its commercial formulations below regulatory limits. Food Chem. Toxicol..

[19] Tarboush N.A., Almomani D.H., Khabour O.F., Azzam M.I. (2022). Genotoxicity of glyphosate on cultured human lymphocytes. Int. J. Toxicol..

[20] Santovito A., Audisio M., Bonelli S. (2020). A micronucleus assay detects genotoxic effects of herbicide exposure in a protected butterfly species. Ecotoxicology.

[21] (2016). EPA’s Off. Pestic. Programs.

[22] International Agency for Research on Cancer (2017). Monographs on the Evaluation of Carcinogenic Risks to Humans.

[23] Bolognesi C., Bonatti S., Degan P., Gallerani E., Peluso M., Rabboni R., Roggieri P., Abbondandolo A. (1997). Genotoxic activity of glyphosate and its technical formulation roundup. J. Agric. Food Chem..

[24] Guilherme S., Santos M.A., Gaivão I., Pacheco M. (2014). Are DNA-Damaging effects induced by herbicide formulations (Roundup® and Garlon®) in fish transient and reversible upon cessation of exposure?. Aquat. Toxicol..

[25] Poletta G.L., Larriera A., Kleinsorge E., Mudry M.D. (2009). Genotoxicity of the herbicide formulation Roundup® (Glyphosate) in Broad-Snouted caiman (Caiman latirostris) evidenced by the comet assay and the micronucleus test. Mutat. Res. Genet. Toxicol. Environ. Mutagen.

[26] Nagy K., Tessema R.A., Budnik L.T., Ádám B. (2019). Comparative Cyto- and genotoxicity assessment of glyphosate and Glyphosate-based herbicides in human peripheral White blood cells. Environ. Res..

[27] Nagy K., Argaw Tessema R., Szász I., Smeirat T., Al Rajo A., Ádám B. (2021). Micronucleus formation induced by glyphosate and Glyphosate-Based herbicides in human peripheral White blood cells. Front. Public Health.

[28] Mesnage R., Ibragim M., Mandrioli D., Falcioni L., Tibaldi E., Belpoggi F., Brandsma I., Bourne E., Savage E., Mein C.A. (2022). Comparative toxicogenomics of glyphosate and roundup herbicides by mammalian stem cell-based genotoxicity assays and molecular profiling in Sprague-Dawley rats. Toxicol. Sci..

[29] Defarge N., Takács E., Lozano V.L., Mesnage R., de Vendômois J.S., Séralini G.E., Székács A. (2016). Co-formulants in Glyphosate-based herbicides disrupt aromatase activity in human cells below toxic levels. Int. J. Environ. Res. Public. Health.

[30] Mesnage R., Antoniou M.N. (2018). Ignoring adjuvant toxicity falsifies the safety profile of commercial pesticides. Front. Public Health.

[31] OECD Test No. 487: In Vitro Mammalian Cell Micronucleus Test,OECD Guidelines for the Testing of Chemicals, Section 4 2023. Available online: 10.1787/9789264264861-en. (accessed on 21 February 2025).

[32] Bolognesi C., Fenech M., Dhawan A., Bajpayee M. (2013). Genotoxicity Assessment: Methods and Protocols.

[33] Makame K.R., Masese S.N., Ádám B., Nagy K. (2023). Oxidative stress and cytotoxicity induced by Co-Formulants of Glyphosate-Based herbicides in human mononuclear White blood cells. Toxics.

[34] Jauregui H.O., Hayner N.T., Driscoll J.L., Williams-Holland R., Lipsky M.H., Galletti P.M. (1981). Trypan blue dye uptake and lactate dehydrogenase in adult rat Hepatocytes--Freshly isolated cells, cell suspensions, and primary monolayer cultures. Vitro.

[35] Zeiger E. (2019). The test that changed the world: the Ames test and the regulation of chemicals. Mutat. Res. Genet. Toxicol. Environ. Mutagen.

[36] Milić M., Kopjar N. (2004). Evaluation of in vitro genotoxic activity of bleomycin and mitomycin c in human lymphocytes using the alkaline comet assay. Arh. Hig. Rada Toksikol..

[37] Bolzán A.D., Bianchi M.S. (2018). DNA and chromosome damage induced by bleomycin in mammalian cells: an update. Mutat. Res. Rev. Mutat. Res..

[38] Cox C., Surgan M. (2006). Unidentified inert ingredients in pesticides: implications for human and environmental health. Environ. Health Perspect..

[39] Mesnage R., Benbrook C., Antoniou M.N. (2019). Insight into the confusion over surfactant Co-Formulants in Glyphosate-based herbicides. Food Chem. Toxicol..

[40] Mesnage R., Bernay B., Séralini G.E. (2013). Ethoxylated adjuvants of Glyphosate-based herbicides are active principles of human cell toxicity. Toxicology.

[41] Aribisala O.A., Sogbanmu T.O., Kemabonta K.A. (2022). Genotoxic, biochemical and histological biomarkers of subacute concentrations of paraquat and glyphosate in Nile tilapia. Environ. Anal. Health Toxicol..

[42] Lal A., Ames B.N. (2011). Association of chromosome damage detected as micronuclei with hematological diseases and micronutrient status. Mutagenesis.

[43] Krupina K., Goginashvili A., Cleveland D.W. (2021). Causes and consequences of micronuclei. Curr. Opin. Cell Biol..

[44] Granada A.E., Jiménez A., Stewart-Ornstein J., Blüthgena N., Reber S., Jambhekar A., Lahav G. (2020). The effects of proliferation status and cell cycle phase on the responses of single cells to chemotherapy. Mol. Biol. Cell.

[45] Luzhna L., Kathiria P., Kovalchuk O. (2013). Micronuclei in genotoxicity assessment: from genetics to epigenetics and beyond. Front. Genet..

[46] Ray J.G., Ranganathan K., Chattopadhyay A. (2016). Malignant transformation of oral submucous fibrosis: overview of histopathological aspects. Oral. Surg. Oral. Med. Oral. Pathol. Oral. Radio..

[47] Santovito A., Nota A., Pastorino P., Gendusa C., Mirone E., Prearo M., Schleicherová D. (2024). In vitro genomic damage caused by glyphosate and its metabolite AMPA. Chemosphere.

[48] Kašuba V., Milić M., Rozgaj R., Kopjar N., Mladinić M., Žunec S., Vrdoljak A.L., Pavičić I., Čermak A.M.M., Pizent A. (2017). Effects of low doses of glyphosate on DNA damage, cell proliferation and oxidative stress in the HepG2 cell line. Environ. Sci. Pollut. Res..

[49] Duarte N. de A.A., Lima L.E. de, Maraslis F.T., Kundi M., Nunes E.A., Barcelos G.R.M. (2021). Acute toxicity and DNA instability induced by exposure to low doses of triclosan and phthalate DEHP, and their combinations, in vitro. Front. Genet..

[50] De Almeida L.K.S., Pletschke B.I., Frost C.L. (2018). Moderate levels of glyphosate and its formulations vary in their cytotoxicity and genotoxicity in a whole blood model and in human cell lines with different estrogen receptor status. 3 Biotech.

[51] European Chemical Agency, E. Registration Dossier (Betaines, C12-14 (Even Numbered)-Alkyldimethyl) Available online: 〈https://echa.europa.eu/registration-dossier/-/registered-dossier/14910/7/4/1〉 (accessed on 18 October 2023).

[52] Woźniak E., Sicińska P., Michałowicz J., Woźniak K., Reszka E., Huras B., Zakrzewski J., Bukowska B. (2018). The mechanism of DNA damage induced by roundup 360 PLUS, glyphosate and AMPA in human peripheral blood mononuclear cells - genotoxic risk assessement. Food Chem. Toxicol..

[53] Hartwig A., Arand M., Epe B., Guth S., Jahnke G., Lampen A., Martus H.J., Monien B., Rietjens I.M.C.M., Schmitz-Spanke S. (2020). Correction to: mode of Action-Based risk assessment of genotoxic carcinogens (archives of toxicology, (2020), 94, 6, (1787-1877), 10.1007/S00204-020-02733-2). Arch. Toxicol..

[54] Principles and Methods for the Risk Assessment of Chemicals in Food; World Health Organization, 2009; ISBN 978-92-4-157240-8. Available online:〈https://iris.who.int/bitstream/handle/10665/44065/WHO_EHC_240_12_eng_Chapter9.pdf;sequence=12〉. (accessed on 6 May 2025).

[55] Gong H., Li R., Li F., Guo X., Xu L., Gan L., Yan M., Wang J. (2023). Toxicity of nanoplastics to aquatic organisms: genotoxicity, cytotoxicity, individual level and beyond individual level. J. Hazard. Mater..

[56] Klátyik S., Simon G., Oláh M., Takács E., Mesnage R., Antoniou M.N., Zaller J.G., Székács A. (2024). Aquatic ecotoxicity of glyphosate, its formulations, and Co-Formulants: evidence from 2010 to 2023. Environ. Sci. Eur..

[57] Barlow S.M., Boobis A.R., Bridges J., Cockburn A., Dekant W., Hepburn P., Houben G.F., König J., Nauta M.J., Schuermans J. (2015). The role of hazard- and risk-based approaches in ensuring food safety. Trends Food Sci. Technol..

[58] Palma-Bautista C., Vazquez-Garcia J.G., Travlos I., Tataridas A., Kanatas P., Domínguez-Valenzuela J.A., De Prado R. (2020). Effect of adjuvant on glyphosate effectiveness, retention, absorption and translocation in lolium rigidum and conyza canadensis. Plants.

[59] European Commission Approval of Active Substances (1487). Safeners and synergists NEW– requirements for safeners and synergists. Off. J. Eur. Union L.

[60] Guilherme S., Santos M.A., Barroso C., Gaivão I., Pacheco M. (2012). Differential genotoxicity of Roundup® formulation and its constituents in blood cells of fish (Anguilla Anguilla): considerations on chemical interactions and DNA damaging mechanisms. Ecotoxicology.

[61] Hao Y., Zhang Y., Cheng J., Xu W., Xu Z., Gao J., Tao L. (2020). Adjuvant contributes Roundup’s unexpected effects on A549 cells. Environ. Res..

[62] Santovito A., Ruberto S., Gendusa C., Cervella P. (2018). In vitro evaluation of genomic damage induced by glyphosate on human lymphocytes. Environ. Sci. Pollut. Res..

[63] Bryce S.M., Avlasevich S.L., Bemis J.C., Phonethepswath S., Dertinger S.D. (2010). Miniaturized flow cytometric in vitro micronucleus assay represents an efficient tool for comprehensively characterizing genotoxicity Dose-Response relationships. Mutat. Res. Genet. Toxicol. Environ. Mutagen.

[64] Koller V.J., Fürhacker M., Nersesyan A., Mišík M., Eisenbauer M., Knasmueller S. (2012). Cytotoxic and DNA-damaging properties of glyphosate and roundup in Human-Derived buccal epithelial cells. Arch. Toxicol..

[65] Ladeira C., Gajski G., Meneses M., Gerić M., Viegas S. (2020). The genotoxicity of an organic solvent mixture: a human biomonitoring study and translation of a real-scenario exposure to in vitro. Regul. Toxicol. Pharmacol..

[66] Wu Q., Liu C., Yang J., Guan A., Ma H. (2017). Design, synthesis, and herbicidal activity of novel quaternary ammonium salt derivatives. Pestic. Biochem. Physiol..

[67] Li B., Li H., Pang X., Cui K., Lin J., Liu F., Mu W. (2018). Quaternary ammonium cationic surfactants increase bioactivity of indoxacarb on pests and toxicological risk to daphnia magna. Ecotoxicol. Environ. Saf..

[68] Mesnage R., Teixeira M., Mandrioli D., Falcioni L., Ducarmon Q.R., Zwittink R.D., Mazzacuva F., Caldwell A., Halket J., Amiel C. (2021). Use of shotgun metagenomics and metabolomics to evaluate the impact of glyphosate or roundup mon 52276 on the gut microbiota and serum metabolome of Sprague-Dawley rats. Environ. Health Perspect..

[69] Ferguson S., Mesnage R., Antoniou M.N. (2022). Cytotoxicity mechanisms of eight major herbicide active ingredients in comparison to their commercial formulations. Toxics.

[70] Panzacchi, S.; Tibaldi, E.; De Angelis, L.; Falcioni, L.; Gnudi, F.; Iuliani, M.; Manservigi, M.; Manservisi, F.; Manzoli, I.; Menghetti, I.; et al. Leukemia in Sprague-Dawley Rats Exposed Long-Term from Prenatal Life to Glyphosate and Glyphosate-Based Herbicides 2023.

